# Architectures for Industrial AIoT Applications

**DOI:** 10.3390/s24154929

**Published:** 2024-07-30

**Authors:** Eneko Villar, Imanol Martín Toral, Isidro Calvo, Oscar Barambones, Pablo Fernández-Bustamante

**Affiliations:** 1 Department of Systems Engineering and Automatic Control, Faculty of Engineering of Vitoria-Gasteiz, University of the Basque Country (UPV/EHU), 01006 Vitoria-Gasteiz, Spain; imanol.martint@ehu.eus (I.M.T.); oscar.barambones@ehu.eus (O.B.); 2 Aeronautical Technologies Centre (CTA), 01510 Miñano Mayor, Spain; 3 Department of Electrical Engineering, Faculty of Engineering of Vitoria-Gasteiz, University of the Basque Country (UPV/EHU), 01006 Vitoria-Gasteiz, Spain; pablo.fernandez@ehu.eus

**Keywords:** Artificial Intelligence of Things, AIoT, reference architectures, industrial applications, Edge/Fog/Cloud Computing, IoT, artificial intelligence

## Abstract

Industry 4.0 introduced new concepts, technologies, and paradigms, such as Cyber Physical Systems (CPSs), Industrial Internet of Things (IIoT) and, more recently, Artificial Intelligence of Things (AIoT). These paradigms ease the creation of complex systems by integrating heterogeneous devices. As a result, the structure of the production systems is changing completely. In this scenario, the adoption of reference architectures based on standards may guide designers and developers to create complex AIoT applications. This article surveys the main reference architectures available for industrial AIoT applications, analyzing their key characteristics, objectives, and benefits; it also presents some use cases that may help designers create new applications. The main goal of this review is to help engineers identify the alternative that best suits every application. The authors conclude that existing reference architectures are a necessary tool for standardizing AIoT applications, since they may guide developers in the process of developing new applications. However, the use of reference architectures in real AIoT industrial applications is still incipient, so more development effort is needed in order for it to be widely adopted.

## 1. Introduction

Industry 4.0 introduced a higher level of digitalization, transforming industrial processes into intelligent, connected, and decentralized production systems [1]. To achieve the goals of Industry 4.0, new technologies such as artificial intelligence (AI) and Industrial Internet of Things (IIoT) are being increasingly adopted in industrial environments [2]. The adoption of these new technologies increases the production efficiency and improves the quality of the products, while at the same time transforming traditional manufacturing processes, making them more sustainable and economically sound [2,3]. The combination of AI techniques with IIoT leads to the creation of a new paradigm, so-called Artificial Intelligence of Things (AIoT) [4]. The combination of AI techniques with IIoT devices in industrial environments helps implement Cyber Physical Production Systems (CPPSs), which are at the core of Industry 4.0. In particular, AIoT improves the monitoring, supervision, predictive maintenance, and control tasks in industrial processes, facilitating the operability, efficiency, and security of production systems.

The emergence of AIoT architectures in the industrial sector is accompanied by other paradigms such as Edge and Cloud Computing. More recently, Fog Computing arose to solve the latency requirements of some applications. Fog Computing creates a bridge between the Edge and Cloud layers where data can be processed and stored locally, reducing latency and increasing flexibility and scalability of industrial systems [5]. Other paradigms similar to Fog Computing have also emerged, such as Mist Computing [6], Cloud of Things, and Cloudlets [7]. All these paradigms and new technologies bring a radical change in the structure and interaction among industrial production systems. Unfortunately, its implementation in real applications may became challenging.

In recent years, designers and developers have increasingly adopted reference architectures as a guide to design and develop their systems, solutions, and application architectures [8]. They define a common vocabulary and provide definitions and design patterns, which may help companies in building their systems and, at the same time, have a huge impact on standardization [9]. Some new reference architectures have been proposed to assist in the process of developing applications [10]. These reference architectures provide new content, vocabulary, structure, and purpose to face the challenges introduced by Industry 4.0 in a huge variety of applications. They are also essential for the establishment of standards in Industry 4.0 and AIoT solutions. Therefore, there is a clear need to find which alternatives exist and understand their purpose, components, and structure. It is also interesting to analyze diverse use cases to find similarities with the new applications [11].

This article investigates the most relevant reference architectures for industrial AIoT applications, analyzing their key characteristics, vocabulary, structure, objectives, and advantages. It also reviews several use cases in order to analyze their applicability for different AIoT applications. This way, a comparison between the existing reference architectures can be carried out, in order to guide researchers in selecting the alternative that best suits each application. We found that existing reference architectures are a necessary tool for guiding the development of AIoT applications and for creating standardized applications in industrial environments. As a result, this review aims at helping developers in building their industrial AIoT applications.

This article will proceed as follows. Section 2 describes the research methodology. Section 3 provides a brief description of Industry 4.0 and AIoT, emphasizing the key concepts and technologies that allow the development of new industrial applications. Section 4 performs a review of the most relevant reference architectures for Industrial AIoT applications and presents use case examples. Section 5 analyzes industrial AIoT applications found in the literature and classifies them into several domains. Section 6 presents a discussion about the research results. Finally, Section 7 draws some conclusions from this review article.

## 2. Research Methodology

Authors considered several key factors for building AIoT applications for industrial environments: AIoT application types, requirements, and needs, Industry 4.0 objectives, and industrial needs for standardization. The following research questions arose:

- **RQ1:** Which reference architectures or standards are available for building industrial AIoT applications?
- **RQ2:** Which reference architecture-based application examples are available in the scientific literature?
- **RQ3:** Which application domains are adopting AIoT-based solutions?

Developing AIoT applications for industrial environments can be challenging for companies and engineers, raising the need of reference architectures focused on achieving Industry 4.0 and AIoT objectives. RQ1 intends to find the most relevant existing reference architectures and standards for designing and building AIoT applications for industrial environments. In this article, the main standard reference architectures are analyzed, describing their key characteristics, vocabulary, and objectives.

The objective of RQ2 is to analyze different applications and use cases in which reference architectures are implemented. These application examples may help developers in choosing the reference architecture that suits their application and offer guidelines for implementing them. In this article, several reference architecture application use cases are reviewed and described in order to help developers find applications that may resemble their application.

RQ3 aims to find different AIoT application examples in the scientific literature. These applications may help developers find AIoT-based solutions for their problems, showing how to develop similar solutions and offering guidelines to implement them. In this article, industrial AIoT applications found in the literature have been described and classified in domains.

The following steps describe the methodology followed. (1) We researched surveys and reviews related to Industry 4.0, Cyber Physical Systems (CPSs), Internet of Things (IoT), and reference architectures for AIoT applications. These studies contain information, descriptions, comparisons, and documentation of existing standard and non-standard reference architectures. (2) We identified the main standard reference architectures (RQ1); the results are shown in Table 1. (3) We conducted research on use case examples of diverse reference architecture implementations (RQ2) whose results can be found in Table 2. (4) We investigated multiple studies related to AIoT applications, which were identified and classified by domain (RQ3); the results of RQ3 can be found in Table 3. Finally, (5) we carried out a critical analysis of the works, identifying the challenges and opportunities that the adoption of these architectures may offer in industrial AIoT applications.

## 3. Background

This section covers the necessary background about important concepts mentioned in this paper: Industry 4.0 and AIoT.

### 3.1. Industry 4.0

Industry 4.0, also known as smart manufacturing or the fourth industrial revolution, was initially proposed in Germany in 2011 [12]. The goals of Industry 4.0 are the following: to provide IT-enabled mass customization of products, to increase the flexibility and scalability of production systems, to improve the supervision and monitoring of manufacturing systems, and to facilitate the communication between devices, sensors, machines, production chains, and corporations [13]. Industry 4.0 increases the productivity, efficiency, safety, and intelligence of production chains, reducing costs and providing automatic smart decision making. Multiple technologies support Industry 4.0, but the following ones are considered as technological enablers, or pillars, of Industry 4.0 [1,14]:

- **Autonomous Robots:** The use of robots in production is evolving in their utility, increasing autonomy, flexibility, and interaction with humans and other robots. Robots offer increased productivity, reduced error rates, more efficient production processes, and the ability to perform high-risk tasks [15].
- **Simulation:** Simulation models improve plant operations by creating virtual models of the factory, also called digital twins [16], which include machines, products and humans [15]. Digital twins are composed of physical and virtual products, and connected data that tie physical products with virtual spaces [17]. The interest in digital twins has grown in recent years due to the advances in related technologies, such as IoT, big data, sensor networks, and data management and processing [18]. The connection between physical and virtual spaces is one of the key challenges for smart manufacturing. Digital twins can integrate the physical and virtual data throughout a product life cycle, which can be used to improve the performance of products and processes in the physical space [19].
- **Horizontal and Vertical system integration:** Horizontal integration combines enterprises and customers in a network of information, management systems, and products to collaborate and compete with each other and become more efficient [20]. Vertical integration consists of the linkage of all the value-added subsystems of a single company. Horizontal and vertical integration makes processes more efficient, reducing costs and producing better products [14].
- **Industrial IoT:** IIoT offers the capability of interconnecting sensors, processes, machines, and industrial systems to the internet and Cloud platforms by integrating smart devices capable of collecting, analyzing, and processing data. The IIoT paradigm increases the flexibility and scalability of industrial systems, offering the ability to monitor, supervise, and connect to Cloud services [21].
- **Cybersecurity:** Industry 4.0 and the IIoT paradigms increase the amount of connected devices, machines, and processes, increasing the need to protect critical industrial systems, manufacturing lines, and communication flows [22].
- **Cloud:** Cloud Computing offers servers with powerful and elastic computing and storage resources. This way, Cloud Computing provides the capability to process and analyze huge amounts of data, easing the development of complex AI-based algorithms [23].
- **Additive Manufacturing:** AM is a process that consists of printing products based on digital designs by depositing successive layers of materials on top of each other [24]. The main benefits of AM are freedom of design, mass customization and waste minimization, and offering the ability to use recyclable materials [25].
- **Augmented Reality:** R is a computer graphic technique where the real world is enriched by an artificial virtual object that includes CAD models, pictures, and symbols [26]. AR can be used in industrial environments to improve work and maintenance procedures by providing virtual guidelines.
- **Big data and analytics:** Big data is the process of collecting and analyzing large datasets from different sources, like sensors, smart devices, and machines. Big data applications are useful for in-process management and productivity improvement in manufacturing processes [27].

Recently, the paradigm of Industry 5.0 has arisen. Industry 5.0 focuses on human–machine collaboration and introduces AIoT-driven technologies like digital twins, Internet of Robotic Things, edge and on-device AI, 5G technologies, and collaborative robot technologies [28,29].

CPSs are at the core of Industry 4.0. CPSs combine communication and control technologies and are responsible for the link between virtual spaces and physical reality [3]. CPSs consist of a control unit able to handle sensors and actuators that interact with the physical world, process the obtained data, and exchange them with other systems or Cloud services using communication interfaces. CPSs are applied in various areas, from manufacturing systems [19,30] to renewable energy applications [31,32], smart buildings [33], and agriculture applications [34]. CPSs in industrial manufacturing and production systems are sometimes called Cyber Physical Production Systems (CPPSs) [35].

### 3.2. AIoT

In recent years, AI-based solutions have been gaining increased popularity in a wide range of applications. Nowadays, machine learning (ML) is the most used method to learn from data, identify patterns, and make decisions [36]. A variety of ML models exist, such as Decision Trees (DT) [37], k-means [38] and Artificial Neural Networks (ANNs). Recently, deep learning (DL) has become the most widely used computational approach in the field of machine learning [39]. DL offers the ability to process, analyze, and learn from massive amounts of data, but it also demands higher-performance computing systems. Convolutional Neural Networks (CNNs) are the most representative DL model [40], but other architectures like Restricted Boltzmann Machine (RBM) and Long Short-Term Memory (LSTM) are also being used [41]. More recently, multiple applications have been using Reinforcement Learning (RL) and Deep Reinforcement Learning (DRL) techniques [42].

The IoT paradigm enables the communication between electronic devices and sensors through the internet, and has become a fundamental technology for developing smart sensors, smart cities, smart homes, smart industries, and healthcare applications [43,44]. Currently, researchers have proposed multiple IoT- and IIoT-based applications for various areas. For example, Behrendt discussed the use of IoT for sustainable forms of transport in smart cities [45]. In [46], Shukla et al. proposed a three-tier architecture for healthcare applications based on the Fog Computing paradigm. Coutinho et al. studied the performance of shared edge for content delivery in smart industry and connected cars [47]. Yang et al. proposed a framework of mobile edge computing-based hierarchical machine learning task distribution for IIoT applications [48]. IoT applications benefit greatly from the introduction of wireless technologies for monitoring and supervision operations, and they also ease the integration of Cloud services.

Cloud Computing offers the possibility of saving, processing, and analyzing huge amounts of data (big data), facilitating the development and implementation of advanced AI and ML services [49]. Although Cloud Computing is a widely used paradigm, it still has some limitations, especially for latency-sensitive cloud-based applications [50]. Fog Computing is a paradigm that introduces local storing and computing capabilities, creating a bridge between the Cloud and Edge layers [51]. Through the combination of these paradigms, a three-layer Edge/Fog/Cloud Computing architecture arises, which offers increased flexibility and scalability, and reduces the latency [52].

In recent years, AI and IoT have been integrated together to form AIoT. The large amount of data acquired by IoT devices offers a great opportunity to train models based on artificial intelligence, especially DL technologies, which greatly help to process and analyze large amounts of data, thus offering the possibility of analyzing complex systems and providing intelligent decision making [4,36]. The integration of architectures based on the Edge/Fog/Cloud Computing paradigm offers great advantages when making AIoT-based applications, since the Edge layer is responsible for obtaining large amounts of data, while the Cloud layer has enough computational capacity to manage large amounts of data and execute sophisticated AI algorithms. AIoT is used to analyze large videos for video surveillance applications [53], in construction engineering tasks [54], in vibration monitoring of rotating machines [55], fish farming [56], and smart home applications [57,58], among others. The work in [59] provides an overview of Industry 4.0 attributes and AIoT devices for smart manufacturing systems.

## 4. Available Reference Architectures

A reference architecture serves as a roadmap for developing system, solution, and application architectures. It establishes uniform definitions for the system under consideration, its subdivisions, and design templates, as well as a common language for discussing implementation specifications and evaluating alternatives. By steering clear of overly specific details, a reference architecture empowers subsequent designs to align with its principles without unnecessary or arbitrary limitations [60]. In recent decades, companies have increasingly adopted reference architectures as a method to develop and standardize their systems. Industry 4.0 completely changes the structure and interconnection of production systems, creating new challenges in the development of Industry 4.0 solutions. In order to achieve Industry 4.0 objectives, new reference architectures are being developed to guide companies and organizations.

In order to ease the selection and implementation of Industry 4.0 reference architectures, many studies reviewed the existing literature and analyzed which are the main reference architectures and their purpose. For example, Kaiser et al. performed a broad review and classification of existing reference architectures for digital manufacturing, identifying several reference architectures, including, RAMI 4.0, IIRA, IMSA, IVRA, IBM Industry 4.0, SITAM, LASFA, 5C, and IoT-RA [61]. This article makes a clear distinction between reference architectures, system architectures, platforms, and frameworks, and proposes a frame of reference for classifying existing reference architectures. An applicability analysis of reference architectures is also performed. Moghaddam et al. discussed the use of RAMI 4.0, IIRA, IBM Industry 4.0, and NIST Smart Manufacturing reference architectures for smart manufacturing applications [8]. This research analyzes how businesses may upgrade their current architectures to meet the characteristics of these smart manufacturing reference architectures and performs a review on service orientation for smart manufacturing. Some other reference architectures are also briefly mentioned, such as IMSA, IVRA, and IoT-RA. In [3], Pivoto et al. performed a survey on the main CPS architecture models. They identify three main reference architectures, 5C, RAMI 4.0, and IIRA. A description of each architecture is presented and their correlation is analyzed, realizing a functional mapping among the three architectures. Standards and protocols that support the implementation of CPS architectures are also detailed. Nakagawa et al. identified and detailed the architectural description of six main reference architectures for Industry 4.0, namely RAMI 4.0, IIRA, IVRA, IBM Industry 4.0, SITAM, and LASFA [11]. These architectures are mapped to the industrial automation pyramid to facilitate their understanding; existing supporting technologies and tools are analyzed. This review briefly mentions other industrial reference architectures like NIST and some reference architectures for IoT applications that can also work in Industry 4.0, such as IoT-A, IoT-RA, and OpenFog.

Helmann et al. performed a review on the literature to find reference architectures that can guide the adoption of new technologies in production systems [62]. The following reference architectures are identified and briefly described: RAMI 4.0, IIRA, IBM Industry 4.0, IMSA, IVRA, and SME. Folgado et al. presented a review on the Industry 4.0 concept, terms, enabling technologies, and reference architectures, in order to guide the design and deployment of automation and supervision systems [63]. The Automation Pyramid and RAMI 4.0 reference architectures are analyzed and described. Other reference architectures, such as IIRA, IMSA, and IVRA, are briefly mentioned. Mirani et al. reviewed RAMI 4.0, IIRA, and OpenFog reference architectures [64]. They made a comparison between these architectures, with a focus on how they approach the development and implementation of industrial IIoT applications. Key IIoT requirements, emerging technologies, and architectures are also analyzed. Weber et al. analyzed IIRA, RAMI 4.0, and SITAM, and extracted features relevant to data-driven manufacturing from these reference architectures. These features are then used to compose six maturity levels and define a model that helps companies assess the maturity of their IT architecture with regard to data-driven manufacturing and Industry 4.0 [65]. Li et al. analyzed smart manufacturing-enabling technologies, architectures, reference models, standards, and frameworks. RAMI 4.0, IMSA, IVRA, IIRA, and SME reference architectures are described, analyzed, and compared [66]. Bader et al. provided a structured analysis of several reference architectures, including IIRA, RAMI 4.0, OpenFog, IVRA, IDS, and IoT-A. These reference architectures are compared and linked to relevant industry standards and technologies [67]. Unal presented a description of some reference architectures, including IIRA, RAMI 4.0, OpenFog, IDS, and IoT-RA [68]. This review also briefly describes some proprietary reference architectures, such as Microsoft Azure IoT RA and IBM Industry 4.0.

Table 1 summarizes the reference architectures described by similar studies. From this literature review, five main reference architectures have been identified, namely RAMI 4.0, IIRA, OpenFog, IMSA, and IVRA. In this section, we describe and analyze each one of these reference architectures, including some application examples that may guide engineers in developing their solutions. Some other reference architectures are also briefly described at the end of this section, since they may also fit in some applications.

**Table 1:** Summary of Industry 4.0 reference architecture studies.

Study	RAMI 4.0	IIRA	OpenFog	IMSA	IVRA	Other RAs
[61]	✔	✔		✔	✔	SITAM SME LASFA IoT-RA IBM-I4.0 5C
[8]	✔	✔		✔	✔	IBM-I4.0
[3]	✔	✔				5C
[11]	✔	✔	✔		✔	SITAM LASFA IoT-RA IoT-A IBM-I4.0
[62]	✔	✔		✔	✔	SME IBM-I4.0
[63]	✔	✔		✔	✔	
[64]	✔	✔	✔			
[65]	✔	✔				SITAM
[66]	✔	✔		✔	✔	SME
[67]	✔	✔	✔		✔	IoT-A IDS
[68]	✔	✔	✔			IDS IoT-RA IBM-I4.0

### 4.1. RAMI 4.0

**Figure 1:**
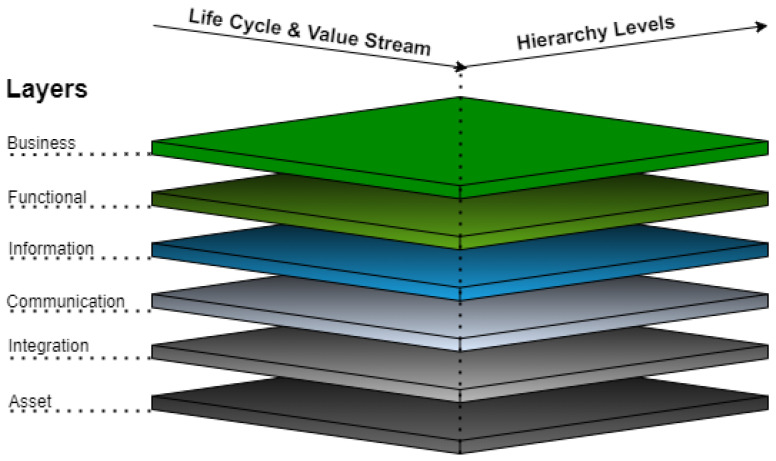
RAMI 4.0 model.

The Reference Architecture Model Industrie 4.0 (RAMI 4.0) was developed by Platform Industrie 4.0 in 2015, with the objective of defining communication structures and a common language within the smart factory [3], enabling the integration of IoT and services in the Industry 4.0 context. RAMI 4.0 is defined by the German standard DIN SPEC 91345:2016-04 [69], and combines aspects related to the manufacturing process, the product, and the IT through a service-oriented three-dimensional hierarchical structure [10]. RAMI 4.0 is divided based on three structural axes, namely layers, life cycle and value stream, and hierarchy levels (Figure 1).

- **Layers:** Layers are used in the vertical axis to represent the various perspectives, such as data maps, functional descriptions, communications behavior, hardware/assets, or business processes. This corresponds to IT thinking where complex projects are split up into clusters of manageable parts.
  – **Business Layer:** Manages, models, and coordinates business functions, rules, and processes in the system. Additionally, this layer ensures the integrity of the functions along the value chain, orchestrates services in the functional layer, and provides a link between different business processes.
  – **Functional Layer:** Represents the environment for describing, integrating, executing, and modelling functions, services, and applications. This layer is also responsible of the horizontal integration of various functions.
  – **Information Layer:** Maintenance, integration, enhancements, and provision of data for the model and functional layer.
  – **Communication Layer:** Standardization and control of communication and data between information and integration layer.
  – **Integration Layer:** This layer is responsible for providing information related to physical resources for higher layers. It contains all the elements associated with IT management and logic control of assets and events related to technology and human interaction.
  – **Asset Layer:** Includes all physical things in the real world, including sensors, actuators, machines and humans. Together with the integration layer, it defines the interface to the real world.
- **Life Cycle and Value Stream:** Industry 4.0 offers great potential for improvement throughout the life cycle of products, machines, and factories. In order to visualize and standardize relationships and links, the second axis of RAMI 4.0 represents the life cycle and the associated value streams. This axis is based in the international standard IEC 62890 [70], which takes care of the life cycle management for systems and products used in industrial processes [71]. This axis is structured in types and instances. A type is created with the initial idea, covering the placing of design orders, development, and testing from the first sample to prototype production. Each manufactured product then represents an instance of that type, having, for example, a unique serial number.
- **Hierarchy Levels:** This axis describes the functional classification of various circumstances within Industry 4.0. For classification within a factory, this axis of the reference architecture follows the IEC 62264 and IEC 61512 standards [72,73]. This axis is divided in seven different hierarchy levels, namely Product, Field Device, Control Device, Station, Work Centers, Enterprise, and Connected World.

RAMI 4.0 is an abstract model that systematizes and structures complex relationships and functionalities required in Industry 4.0 applications. From a technological point of view, RAMI 4.0 is a generic and neutral model, not an implementation guide, and does not provide support for the practical development of Industry 4.0 applications. As a result, many authors have studied and analyzed the requirements of developing RAMI 4.0-compatible solutions and proposed application examples and guidelines for facilitating their implementation [74]. For example, López et al. proposed a platform aligned with RAMI 4.0 that offers tools and resources to facilitate the development of Industry 4.0 components [75]. In [71], Melo et al. developed an open-source control device for Industry 4.0 applications based on the RAMI 4.0 model. Authors in [76] demonstrated the applicability of RAMI 4.0 concepts and technologies to a system for concurrent product design and assembly planning. Contreras et al. retrofitted their system based on RAMI 4.0 for the correct implementation of Industry 4.0 applications [77]. Lins et al. developed a platform based on RAMI 4.0 that supports the standardization of a retrofitting process to transform old equipment into a CPPS [78]. Schulte et al. described the development of an industrial plastic plate extruder system based on RAMI 4.0 [79]. The platform is validated with an industrial robotic arm prototype. In [80], Ye and Hong proposed a four-layer manufacturing system architecture based on RAMI 4.0. The architecture is validated in a manufacturing system prototype using AML and OPC UA technologies. Pisching et al. proposed a platform to discover equipment to process operations according to the product requirements based on RAMI 4.0 [81].

To achieve industry interconnection and facilitate cooperation between different enterprises, it is essential to analyze the possible alignment and cooperation between standard reference architectures for Industry 4.0. For this reason, in 2015, the Sino-German Standardization Cooperation Commission was set up in order to perform an alignment between RAMI 4.0 and IMSA reference architectures [82]. A similar collaboration has been made between the Industrial Internet Consortium (IIC) and Platform Industrie 4.0 to explore the potential alignment of RAMi 4.0 and IIRA, understand the technical issues from both perspectives, and reduce market confusion [83]. Other studies have also analyzed the possible alignment between existing reference architectures. For example, Fraile et al. provided research on prominent reference models for IIoT systems [84]. This article analyzed NIST-SME, IMSA, and IIRA, and performed an alignment between these architectures and RAMI 4.0. Based on their analysis, they proposed an Industrial Internet Integrated Reference Model. Nazarenko et al. provided an analysis of relevant standards for manufacturing systems and aligned those standards with the RAMI 4.0 reference model [85].

### 4.2. IIRA

The Industrial Internet Reference Architecture (IIRA) is a standard-based open architecture developed by the Industrial Internet Consortium (IIC) that is applied to the Industrial Internet domain. IIRA has a broad industry applicability, supported by a generic description and representation at a high level of abstraction, to enhance common understanding, drive interoperability, map applicable technologies, and guide technology and standard development [60]. IIRA offers guidelines to provide assistance and guidance for the development, documentation, communication, and deployment of IIoT systems, and is continually refined with feedback gathered from applications developed by IIC and from real-world deployments. Also, IIRA can be systematically used as an architectural template to define the specific requirements and designs of each IIoT system. The IIRA architecture consists of four viewpoints, the Enterprise viewpoint, Usage viewpoint, Functional viewpoint, and Implementation viewpoint:

1. **Enterprise viewpoint:** Identifies stakeholders and their goals, values, and objectives to establish an IIoT system within their enterprise and regulatory context.
2. **Usage viewpoint:** Describes the use of the system as user activities that achieve the system’s capabilities.
3. **Functional viewpoint:** Defines the functional components of the IIoT system and how they relate to the environment and the system.
4. **Implementation viewpoint:** Assists in choosing the technologies, components, and communications of the IIoT system.

**Figure 2:**
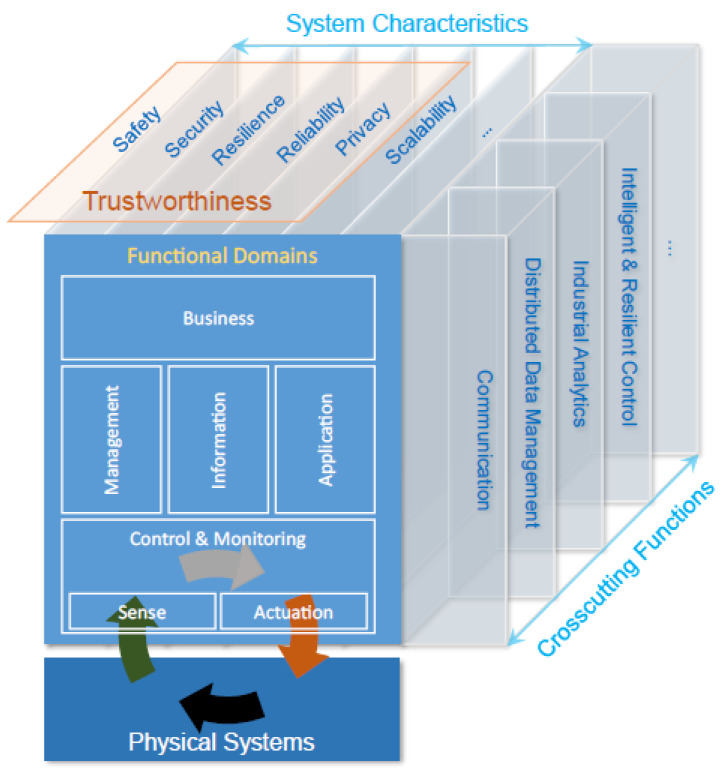
IIRA functional domains, system characteristics and crosscutting functions [60].

IIRA decomposes the typical IIoT systems into five functional domains, namely, Control and Monitoring Domain, Information Domain, Application Domain, Business Domain, and System Management Domain. This functional domain’s component focuses on major system functions that are required to support generic IIoT usages and to realize generic IIoT system capabilities for business purposes. In Figure 2, the relationships between the functional domains, crosscutting functions, and key system characteristics are summarized.

- **Control and Monitoring Domain:** This functional domain contains the functions performed by industrial control and automation systems. The core functions of this domain include reading data from sensors, exercising control over the physical systems through actuators, and enabling the information exchange between the system components.
- **System Management Domain:** This domain manages the functional components of complex, loosely coupled, and distributed IIoT systems.
- **Information Domain:** This domain contains a collection of functions for gathering data from various domains, especially from the control domain. The information domain also handles data processing, collection, and performs data analytics.
- **Application Domain:** This domain contains functions for implementing application logic. These functions implement application logic, rules, and models for high-level optimization. It also includes APIs and UIs, making the relevant information available for human interaction.
- **Business Domain:** Supports business processes and procedural activities that an IIoT system must integrate to enable end-to-end operations. Some of these business functions are Enterprise Resource Planning (ERP), Product Life cycle Management (PLM), Manufacturing Execution System (MES), billing and payment, work planning, and scheduling systems.

Many authors have studied and developed applications based on IIRA in different scenarios. For example, Baudoin proposed a roadmap for Industrial Internet applications based on IIRA for oil and gas [86]. Morkevicius described a mapping of IIRA to UAF and provided a case study to show the application of the UAF for modeling IIoT architectures [87]. Koncoro et al. designed an Information Management System based on the IIRA model [88]. Alonso-Perez et al. applied IIRA to the research and development of the production of thin-film photovoltaic modules [89]. Leitao et al. provided insights related to the use and alignment of IEEE 2660.1 recommended practices to support CPS developers and engineers in integrating assets in the context of RAMI 4.0 and IIRA reference models [90]. In [91], da Rocha et al. integrated the IEEE 1451 and IEC 61499 standards [92,93] with the IIRA model and developed a case study representing a car painting line in a production plant. Melluso et al. proposed an approach that enhances interoperability between Industry 4.0 standards, such as IIRA and RAMI 4.0 [94]. Pedone and Mezgár compared IIRA and RAMI 4.0 frameworks in the context of Cloud Computing and provided an example of how manufacturing services can be conceptualized and orchestrated in each architecture [95].

### 4.3. OpenFog

**Figure 3:**
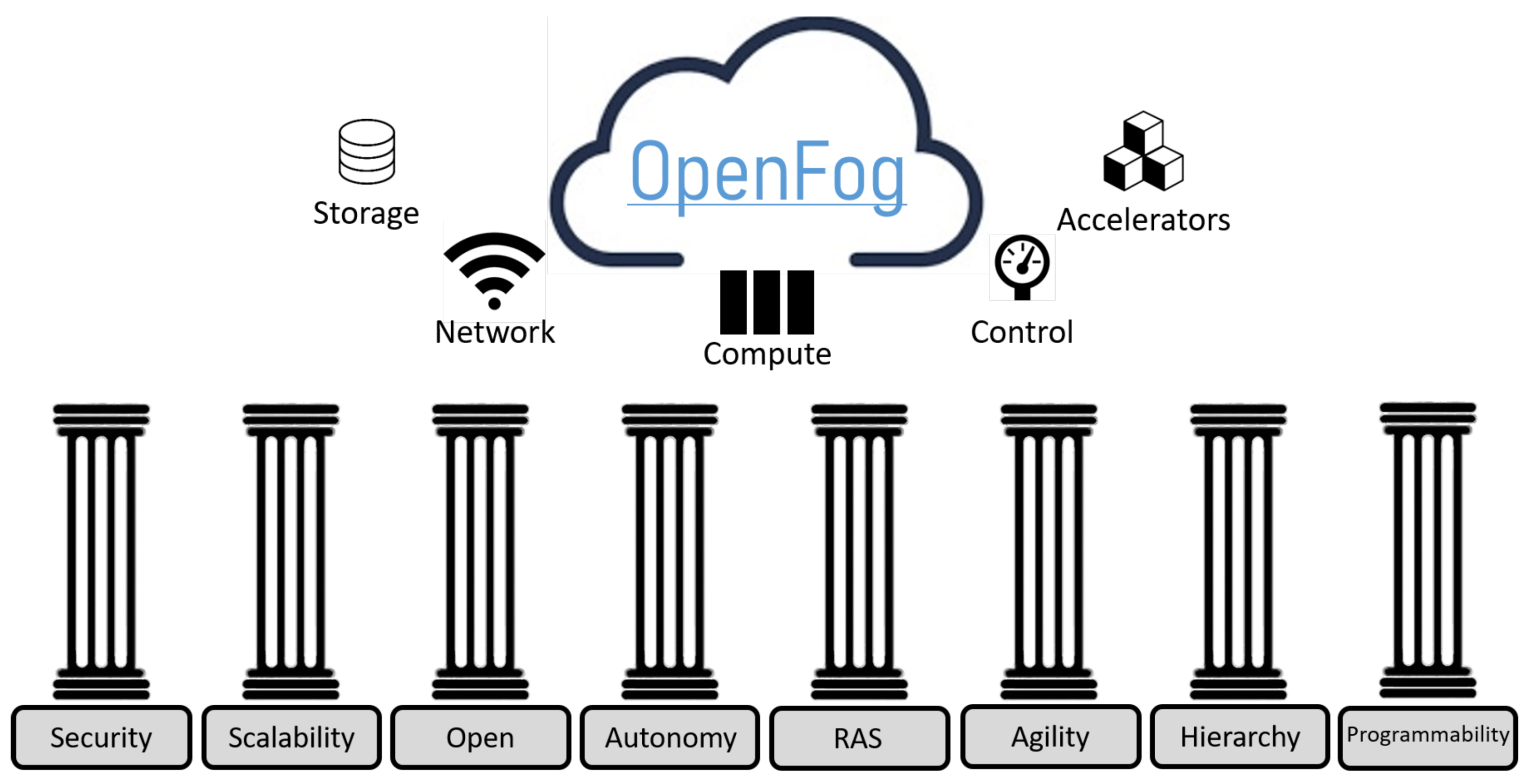
Pillars of OpenFog reference architecture [97].

The OpenFog Consortium, established in 2015 with founding members such as ARM, Cisco, Dell, Intel, Microsoft, and Princeton University, aims to standardize the implementation of Fog and Edge Computing technologies. OpengFog’s approach focuses on enabling efficient communication between Fog–Fog and Fog–Cloud Tiers. This approach offers benefits such as improved security, cognitive capabilities, and agility, reduced latency, and enhanced efficiency, which are refereed using the SCALE acronym. The IEEE 1934-2018 OpenFog standard emerged as a result of this initiative. The OpenFog Reference Architecture guidelines include additional security to ensure secure and trusted transactions, customer-centric goal awareness to enable autonomy, agility for rapid innovation, and affordable scalability under a common infrastructure, latency for real-time processing and cyber physical system control, and efficiency for dynamic pooling of unused local resources from participating end-user devices [96]. OpenFog consists of eight main pillars that define the architecture. These pillars represent the key attributes that must be satisfied to embody the OpenFog definition of a horizontal, system-level architecture that provides the distribution computing, storage, control, and networking functions closer to the data source along the cloud-to-thing continuum. These pillars and their objectives are shown in Figure 3.

1. **Security:** Security is one of the priorities of OpenFog RA, with adaptable measures of privacy, integrity, and trust. Compliance ensures end-to-end security with hardware-based trust roots.
2. **Scalability:** The architecture scales internally, within networks, and elastically. It adapts to the needs and resources of the fog application.
3. **Openness:** Promotes diversity and innovation through interoperability, versatility, and transparency.
4. **Autonomy:** Reduces dependency on the Cloud, enabling efficient and context-aware data decisions and transmission with the DIKW model and AI.
5. **RAS (Reliability, Availability, Serviceability):** Ensures functionality, reliability, fault detection, redundancy, and automation.
6. **Agility:** Enables data-driven IoT decisions and dynamic fog deployments, reducing network dependencies and optimizing application placement.
7. **Hierarchy:** Provides scalable and flexible computing resources for IoT needs. Fog nodes operate autonomously and self-manage.
8. **Programmability:** Programmability allows for automated function reassignment, offering flexibility, resource efficiency, multitenancy support, cost-effective operations, and enhanced security.

OpenFog shows the software and hardware structure on which the above concepts are supported. They can be defined in three concepts:

- **Software View:** This perspective is depicted in the top three layers of the architectural description, covering Application Services and Support, Node Management, and the Software Backplane.
- **System View:** System View is represented in the intermediate layers of the architectural description, spanning from Hardware Virtualization to Hardware Platform Infrastructure.
- **Node View:** This perspective is represented in the two lower layers and includes the Protocol Abstraction Layer and Sensors, Actuators and Control systems.

In the scientific literature, some OpenFog reference architecture-based application examples and frameworks can be found. For example, Modarresi et al. defined a framework based on the OpenFog reference architecture that integrates software-defined networking with fog nodes to improve the resilience of networks [98]. In order to validate the proposed framework, they developed and tested an IP spoofing security application in a fog node. Kuo et al. proposed a framework for the integration of Edge and Fog Computing and networking based on the OpenFog and ETSI MEC ISG specifications [99]. ETSI MEC and OpenFog Consortium have reached an understanding, with the intent to share work related to global standards development for fog-enabled mobile Edge technologies and applications. Muneeb et al. developed an OpenFog-based multilayered architecture for latency-sensitive data analysis between Cloud and Fog Computing layers [100]. The proposed architecture was validated through a case study of a camera surveillance application. Gebremichael et al. analyzed the requirements specified for secure and private IIoT applications in industry standards, such as Industrial Internet Consortium (IIC) and OpenFog Consortium [101]. This article also discusses future research directions to enhance security, privacy, and safety of IIoT applications. Toral et al. proposed an open multilayer architecture for smart buildings based on the OpenFog reference architecture [97]. The proposed architecture is experimentally validated through an AIoT application to improve indoor environmental quality using Fuzzy logic. Yanuzzi et al. proposed a converged model that combines the strengths of OpenFog and ETSI MANO architectures and applies their combined capability for IoT applications [102]. The proposed paradigm is validated through real examples for implementing uniform security for industry and smart city applications. Dlamini et al. proposed an enhancement to autonomous management and orchestration capabilities of Fog Computing networks [103]. This framework combines OpenFog and ETSI NFV MANO architectures and adds a finite state machine component to enhance decision making in the edge node of the network. Beraldi and Alnuweiri proposed a distributed Fog-to-Fog cooperation algorithm based on OpenFog that allows for sharing computation resources among fog providers that agree on a measure of fairness [104].

### 4.4. IMSA

In 2015, the ministry of Industry and Information Technology (MIIT) and Standardization Administration of China (SAC) jointly formulated the Guideline for the Construction of the national Intelligent Manufacturing Standards Systems (Version 1.0) [105], where the Intelligent Manufacturing Systems Architecture (IMSA) was described. Since then, new versions of this document were published in 2018 (Version 2.0) and 2021 (Version 3.0), the latter of which is currently the newest version available, with the objective of improving the IMSA standard, meeting the needs of technological progress and intelligent manufacturing development, and guiding the construction of standard intelligent manufacturing systems for all relevant industries [106].

**Figure 4:**
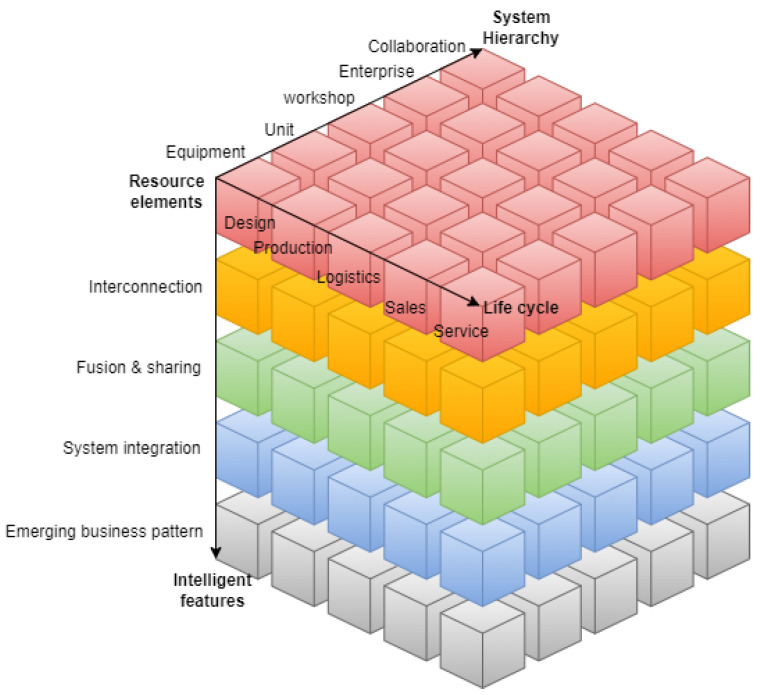
Intelligent Manufacturing System Framework (IMSA).

IMSA describes the activities, equipment, and characteristics involved in intelligent manufacturing from three different perspectives, namely Life Cycle, System Hierarchy, and Intelligent Features [106]. This way, IMSA describes a three-dimensional Intelligent Manufacturing System Framework used to define standardization demands, objects, and scope of intelligent manufacturing (Figure 4).

Life cycle perspective includes a series of connected value creation activities, from the product prototype research and development, to product recycling and re-manufacturing. The activities throughout the life cycle, namely Design, Production, Logistics, Sales, and Service, can be optimized iteratively and in a sustainable way.

- **Design:** Process of realizing and optimizing the demands according to the enterprise constraints and the selected technologies.
- **Production:** Processing, transporting, assembling, and inspecting materials to create new products.
- **Logistics:** Physical flow of process of goods from the supplying place to the delivery place.
- **Sales:** Business activities of products or commodities transferred from enterprises to customers.
- **Service:** Activities and results generated throughout the interaction between the product provider and the customers.

The System Hierarchy perspective divides the organizational structure related to the enterprise production into different hierarchy levels, namely the Equipment Level, Unit Level, Workshop Level, Enterprise Level, and Collaboration Level.

- **Equipment Level:** Level where the physical process is realized, perceived, and controlled, using different sensors, instruments, meter, machines, and devices.
- **Unit Level:** Level used to process information, and monitor and control physical processes.
- **Workshop Level:** Production management for the workshop or the factory.
- **Enterprise Level:** Level of enterprise operation and management.
- **Collaboration Level:** Business collaboration, interconnection, and sharing of internal and external information between enterprises.

The Intelligent Features perspective refers to the representation of self-sensing, self-decision making, self-execution, self-learning, self-adaptation, and other functions of manufacturing activities. The Intelligent Features include five levels, namely, Resource Elements, Interconnection, Fusion and Sharing, System Integration, and New Business Pattern.

- **Resource Elements:** Resources or tools that enterprises use in production and the level of their digital model.
- **Interconnection:** Level of data transfer and parameter semantic exchanges between resource elements, through wired or wireless networks, communication protocols, and interfaces.
- **Fusion and Sharing:** Level of information collaborative sharing based on interconnection.
- **System Integration:** Level of data exchange and functional interconnection among equipment, production units, production line, digital workshop, and smart factory, as well as among intelligent manufacturing systems in the process of realizing intelligent manufacturing.
- **Emerging Business Pattern:** Level which covers the functions of cognition, diagnosis, prediction, and decision making, supporting the virtual–real iterative optimization on the basis of data, models, and systems, integrated and fused by the resource elements of different levels in physical space and digital space.

The key of intelligent manufacturing is to achieve vertical, horizontal, and end-to-end integration: vertical integration through the Equipment, Unit, Workshop, Enterprise, and Collaboration levels, horizontal integration across Resource Elements, Interconnection, Fusion and Sharing, System Integration, and Emerging Business Pattern, and end-to-end integration of Design, Production, Logistic, Sales, and Service [82].

Some application examples of the IMSA architecture can be found in the scientific literature. For example, Wei et al. illustrated the essential elements of IMSA and presented two use case examples [107]. The first example presents a Haier Group interconnected factory application, and the second one develops an industrial internet-based monitoring and management system for distributed bioenergy generation. In [82], they analyzed three use cases and compared the implementation of IMSA and RAMI 4.0 architectures. These use case applications include the development of a non-contact radar detector sensor, energy saving in a cooling system, and equipment life cycle management.

### 4.5. IVRA

The Industrial Value Chain Reference Architecture (IVRA) is a reference architecture proposed by the Industrial Value Chain Initiative (IVI), a forum founded in Japan in 2015 to promote smart manufacturing based on the concept of “loosely defined standard” [108]. IVRA responds to the needs produced by the introduction of IoT-based technologies in industrial environments, where manufacturing processes and IT are rapidly merged. This architecture offers a guide and methodology for the design, implementation, and evaluation of intelligent systems, promoting interoperability, modularity, scalability, and safety, with the objective of encouraging innovation and development of intelligent systems, fostering research, experimentation, and standardization. The IVRA defines three independent layers of manufacturing, namely the Business Layer, Activity Layer, and Specification Layer:

- **Business Layer:** This layer focuses on the management of business strategies, inter-company business transactions, and products or services. This layer is represented as a cube composed of three views, namely the Asset View, Activity View, and Management View.
- **Activity Layer:** This layer includes all concrete activities performed by people, machine processes, and software, and the information obtained from such activities and processes.
- **Specification Layer:** In this layer, engineering is executed to transmit, process, and reuse knowledge and know-how, declaring and modelling the contents of the actual production mechanisms.

**Figure 5:**
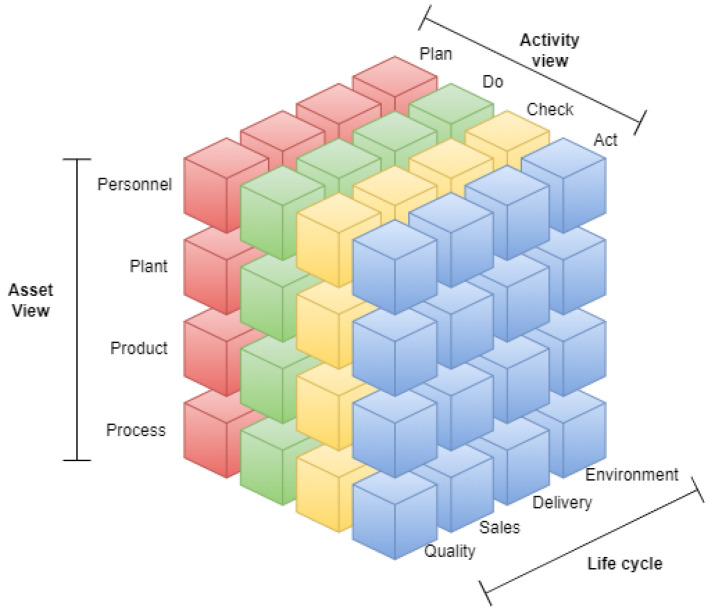
Smart Manufacturing Unit (IVRA).

IVRA defines a Smart Manufacturing Unit (SMU) as an autonomous body that conducts smart manufacturing. SMUs consist of three views (Figure 5), namely the Asset view, Activity view, and Management view:

- **Asset View:** The asset view of an SMU shows assets valuable to the manufacturing organization. In this view, there are four classes of assets: Personnel Assets, Plant Assets, Product Assets, and Process Assets.
  – **Personnel Assets:** Personnel working at production sites conducting operations such as producing products, making decisions, and giving instructions.
  – **Plant Assets:** Equipment, machines, and devices used for manufacturing products, including necessary equipment such as jigs, tools, and subsidiary materials.
  – **Product Assets:** Products created as an outcome of manufacturing and materials used during production are assets.
  – **Process Assets:** Knowledge of operations such as production processes, methods, and know-how.
- **Activity View:** The activity view covers activities performed by SMUs at manufacturing sites in the real world, which can be viewed as a dynamic cycle continuously improving targeted issues proactively. The activity view is composed of the cycle of four key activity classes: “Plan”, “Do”, “Check”, and “Act”.
- **Management View:** The management view shows purposes and indices relevant for management. Assets and activities of SMUs should be appropriately steered in terms of Quality, Cost, Delivery, and Environment, which represent the management view.
  – **Quality:** Quality is an index to measure how the characteristic of a product or service provided by an SMU serves the needs of customers or the external world.
  – **Cost:** Cost is understood as the sum of financial resources and goods spent directly and indirectly in order for an SMU to provide a certain product or service.
  – **Delivery accuracy:** Delivery accuracy is an index showing whether or not the time required to deliver the product or service meets the needs of the SMU’s customers.
  – **Environment:** Environment is an index measuring the degree to which an SMU’s activities harmonize with the environment without placing an excessive strain on the natural world.

Nishuioka et al. mentioned various use case applications developed in the first annual cycle of IVI in 2015 [109]. These use cases include applications such as a Cloud-enabled monitoring platform for global distributed factories, cyber physical production and logistics systems with a common interface, interoperable life cycle management for equipment and production line, real-time sensor data acquisition and analysis using a multivendor network, and mass-customization for an end user directory connected to factories. More information and application example white papers can be found on IVI’s website [110].

### 4.6. Other Architectures

Currently, multiple reference architectures are being developed for AIoT, CPS, IoT, or smart industry applications. Some of these reference architectures may become more relevant in the future with the development of new technologies and paradigms. For this reason, in this section, some less relevant reference architectures are briefly described and use case applications are presented. Table 2 summarizes the documentation and application examples found in the scientific literature of each one of the described reference architectures.

**Table 2:** Summary of reference architecture application examples.

Reference Architecture	Documentation	Examples
RAMI 4.0	[69]	[71,74,75,76,77,78,79,80,81,82,90,94]
IIRA	[60]	[86,87,88,89,90,91,94,95]
OpenFog	[96]	[97,98,99,100,101,102,103,104]
IMSA	[106]	[82,107]
IVRA	[108]	[109,110]
5C	[111]	[112,113,114,115,116]
LASFA	[117]	[118,119,120,121]
SITAM	[122,123]	[124]
IoT-RA	[125]	[126]

Lee et al. proposed the 5C Architecture in 2015 as a guide to develop and implement CPSs in industrial environments [111]. The 5C Architecture defines five levels, namely the Smart Connection Level, Data-to-Information Conversion Level, Cyber Level, Cognition Level, and Configuration Level. The Smart Connection Level focuses on acquiring accurate and reliable data from sensors, controllers, machines, or enterprise manufacturing systems such as ERP, MES, SCM, and CMM. In the Data-to-Information Conversion Level, meaningful information is obtained from the collected data. This level includes mechanisms for prognostics and machine health management applications, which bring self-awareness to machines. The Cyber Level acts as the central information hub of the architecture, gathering information from every machine in the machine network. This level manages and analyzes the collected information to obtain additional information about the status of individual machines, realize performance comparisons, and predict the future behavior of machinery. In the Cognition Level, collected information and knowledge are presented to users for making decisions about task priority and optimization of the maintaining processes. The Configuration Level gives feedback from the cyber space to the physical space and acts as supervisory control to make machines self-configured and self-adaptive.

Some 5C Architecture application examples can be found in the scientific literature. In [112], Ahmed developed an autonomous landing guidance assistance system based on 5C Cyber and Cognition Levels to increase the reliability of the vertical landing mechanism of an urban air mobility vehicle. Fenza et al. proposed an integration of Semantic Web models for implementing a 5C architecture [113]. Authors in [114] designed a smart vision sensor based on the 5C architecture for evaluating a machine surface during the cutting process. Shi et al. presented 5G wireless communication technologies and discussed how to implement these technologies for developing collaborative intelligent manufacturing systems and processes based on the 5C Architecture [115]. Xu et al. presented a collision-free fuzzy formation control method of swarm robotic CPSs using a robust orthogonal firefly algorithm [116]; each mobile robot is implemented using the 5C architecture and communicates by using wireless networks.

Based on the 5C Architecture, Jiang proposed the 8C Architecture [127]. The 8C Architecture extends the 5C Architecture, defining 3C facets (coalition, customer, and content) that emphasize the vertical and horizontal integration of the CPS and include the customer in the manufacturing process. The coalition facet focuses on the value chain and production chain integration among the different parties involved in the production process. The customer facet focuses on the role of the customer in the design process, production processes, and after-sales service of the product. The content facet focuses on extracting, storing, and inquiring about the production information (material suppliers and production processes, parameters, and shipment) and after-sales service details (product maintenance, parts replacement, recycling, and client suggestion, complaints, or comments). Ahmadi et al. compared 5C, 8C, and ACPS reference architectures, and proposed an enhanced 3C CPS architecture for smart manufacturing systems [128].

Resman et al. proposed LASFA (LASIM Smart Factory) in 2019 as a simple model for implementing Industry 4.0 key technologies for smart factories [117]. The LASFA architectural model is based on RAMI 4.0 and focuses on the communication between systems in the smart factories. As explained in detail in Section 4.1, RAMI 4.0 is a generic and standard-based reference architecture that offers an overview of the key technologies of Industry 4.0. The LASFA architectural model is more specific and offers a simple visualization of the entire architecture of the smart factory. This architecture defines the exact locations and functions of different technologies, making it easier to understand and implement in industrial environments. Some application examples of LASFA can be found in the literature. Jankovič et al. implemented artificial intelligence in the concept of a hydraulic press with regard to I4.0 technologies [118]. The hydraulic press is integrated as a CPS into the framework of a smart factory based on the LASFA architectural model. Resman et al. proposed an approach for developing data-driven digital twins of manufacturing systems and processes based on LASFA [119]. Sun et al. proposed an extension to the LASFA framework called LASFA+ [120]. LASFA+ gives a wider perspective to the elements that participate in production, providing complementary information to the production process. The proposed architecture was validated by implementing the logistics of the delivery of industrial components in the construction sector, where different stakeholders benefit from the enhanced shared knowledge provided by LASFA+. Ordieres-Meré et al. proposed a flexible platform under the LASFA+ reference framework [121]. This platform focuses on facilitating the decision-making process on the basis of an extended understanding of the events in the production process, including those impacted by human operators. In order to demonstrate the advantages of the proposed platform, two applications were developed. The first application implements inner logistics in a rebar factory, and the second one focuses on ergonomics and process variability in an automotive component supplier.

Authors in [122,123] proposed Stuttgart IT Architecture for Manufacturing (SITAM), a conceptual IT architecture that enables companies to realize and implement a data-driven factory. This architecture encompasses the entire product life cycle, from processes and physical resources to IT systems and web data sources. The SITAM architecture describes the following components: the integration middleware, the analytics middleware, the mobile middleware, service composition and value-added services, and cross-architectural topics. The integration middleware provides flexibility and adaptability to manufacturing companies, offering services, data exchange formats, and mediation and orchestration functionalities. The analytics middleware comprises several manufacturing-specific analytics components for a data-driven factory. The mobile middleware enables mobile information provisioning and data acquisition to develop and integrate manufacturing-specific mobile applications. The mobile middleware and analytics middleware are built upon the integration middlware to enable the composition of value-added services for human users and machines. The added value from these services feeds back into the product life cycle for continuous proactive improvement and adaptation. Cross-architectural topics represent overarching issues relevant for all components and comprise data quality, governance, security, and privacy. Königsberger and Mitschang presented the concept and prototype of an SOA Governance Repository (SGR) [124]. SGR is described as a central tool to manage and govern all SOA-related activities within a company, which is an integral part of the SITAM architecture in realizing the data-driven factory. An API is included in the SITAM Architecture to access service endpoint information.

IoT-RA provides a standardized IoT reference architecture, defining a common vocabulary, the main characteristics of IoT applications, and a scalable design. This reference architecture is based on the ISO/IEC 30141 standard [125] and provides examples of best practices for industrial IoT applications. IoT-RA is described by four views, namely the functional view, system deployment view, networking view, and usage view. The functional view describes the distribution and dependencies for supporting activities described in the usage view, addressing domain functions and cross-domain capabilities. The system deployment view describes the generic components, including devices, subsystems, and networks to form an IoT system. The IoT RA networking view describes the principal communications networks which are involved in IoT systems and the entities with which they connect. The usage view focuses on how the IoT system is developed, tested, operated, and used from a user perspective. In [126], an application example of IoT-RA applied to the smart home domain can be found. This article also includes guidelines on how to implement IoT reference architectures.

The NIST (National Institute of Standards and Technology) have proposed various architectures that may be applied for the development of smart manufacturing systems. Some of these architectures include the NIST SME (Smart Manufacturing Ecosystem) architecture [129], NIST SOA (Service-Oriented Architecture) [130], and NIST Framework for Cyber-Physical Systems (NIST F-CPS) [131]. Some reference architectures have been developed through European research projects. For example, a reference architecture for IoT applications called IoT-A (Internet of Things Architecture) or IoT-ARM (Internet of Things Architectural Reference Model) was developed [132], in addition to the Industrial Data Space (IDS) reference architecture [133], which is currently promoted by the Industrial Data Space Association (IDSA). Some researchers have also developed their own architecture, for example, FECIoT [134], UAF1.0 [87], RAMEC [135], FECIoT [134], and Sophon Edge [23].

IBM published a vendor-specific Industry 4.0 Reference Architecture as their proposal to develop Industry 4.0 solutions for manufacturing processes [136]. This architecture includes guidelines to improve performance, scalability, maintainability, availability, security, manageability, usability, and data volumetrics. Performance guidelines refer to the speed and efficiency of the system in executing tasks. Scalability focuses on the system’s ability to handle growing workloads and data. Maintainability guidelines focuse on facilitating the modifications and reparations of a system. Availability refers to the likelihood of the system being operational and accessible. Security focuses on the protection of the system and data against threats and attacks. Manageability focuses on the ease of managing and monitoring an industrial system, process, or machine. Usability focuses on facilitating the use and understanding of the system by its users. Data volumetrics refers to the dimensions and characteristics of the data that the system need to store and analyze. The IBM Industry 4.0 Architecture proposes three layers for describing the functional architecture of a manufacturing system: the Edge Layer, Plant Layer, and Enterprise Layer. The Edge Layer is responsible for connecting sensors, devices, and machines in the production plant and performing real-time analysis and actions at the network edge. The Plant Layer is responsible for integrating data, processes, and services in the production plant and performing cognitive analysis and optimization in the private or hybrid Cloud. The Enterprise Layer is responsible for connecting data, processes, and services in the enterprise and performing business analysis and collaboration in the public or hybrid Cloud. The foundation of this architecture includes key considerations such as intelligence, automation, customization, and innovation, among other aspects. Further information about the IBM Industry 4.0 architecture, products, and solutions can be found on IBM’s website [137].

## 5. Industrial AIoT Application Domains

Nowadays, multiple studies are being published by different researchers regarding AIoT applications. These studies offer guidelines and frameworks for the development of AIoT-based solutions for different industrial systems. In this section, we summarize industrial AIoT applications found in the scientific literature and classify them into different domains. This way, developers may find solutions and guidelines for applications that are similar to their problems. This review may also help developers find out what kind of AIoT applications are currently being developed and how they can be implemented.

In order to perform a classification of AIoT studies, we took inspiration from both “Table 1” in [1] and “Table 8” in [2], in which several studies are analyzed and their applications are described. From this analysis and examining the main technologies used in the selected AIoT studies and their purpose, several application domains have been identified. The main technologies found are digital twin, augmented reality and artificial intelligence techniques, which are part of Industry 4.0, analyzed in Section 3. AI techniques have been classified into two principal domains, namely classification and optimization. The other domains focus on the purpose of the application developed in the study, which are mainly Control, Energy Efficiency, Security, Maintenance, and Signal Processing.

Table 3 summarizes some industrial AIoT applications addressing the identified domains. It follows a short description of these works. Liu et al. proposed an AIoT-empowered Edge–Cloud collaborative computing system for developing an energy-efficient low-latency face tracking application [138]. They developed an FPGA-based Convolutional Neural Network (CNN) accelerator to ensure low latency and they conducted experiments to evaluate the energy cost and execution time of CNN in the face tracking systems. This article developed an AI-based face tracking application, which is a classification AI technique.

**Table 3:** Summary of AIoT application domains.

Application Domain	Studies
Digital Twin	[56,139]
Energy efficiency	[140,141]
Optimization	[56,139,142,143]
Security	[53,140,144,145]
Control	[55,56,99,139,141,142,146]
Maintenance	[55,143,147]
Augmented Reality	[141,147]
Classification	[53,56,138,143,144,146,148]
Signal Processing	[149]

Mian et al. proposed an AIoT-based framework for anomaly detection in rotating machines through vibration monitoring. This framework allows for the remote control and monitoring of the machines in real time using an Edge-centric mechanism based on support vector machine and a dedicated web-based platform [55]. This study combines control strategies and AI-based predictive maintenance of rotating machines. Ullah et al. proposed an efficient and robust AIoT-based framework for recognizing anomalies in large volumes of surveillance video data for smart city and smart factory applications. The ongoing events are classified as normal or anomalous by Convolutional Neural Networks [53]. This article provides security measures using classification AI-based techniques. Ubina et al. presented the design of an AIoT-based digital twin infrastructure for intelligent fish farming [56]. The proposed infrastructure offers services for automated fish feeding, metric estimation (including fish count, size, weight, and species classification), and environmental and health monitoring. This article combines digital twin with AI techniques for fish species detection (classification) and production strategy optimization using particle swarm algorithm. Wang et al. proposed an AIoT-based intelligent signal processing method based on CNN against impulsive noise interference produced by large mechanical and electrical equipment used in coal mines [149]. Zhang et al. proposed a blockchain-empowered AIoT framework that achieves flexible and secure Edge service management [145]. This article focuses in providing secure data management and communication. Fernández-Caramés et al. proposed an augmented reality architecture based on cloudlets and the Fog Computing paradigm [147]; it was evaluated in a real-world scenario using Microsoft HoloLens for remote guidance. The developed augmented reality application has been validated by performing maintenance procedures on bending machines. Suárez-Albela et al. provided an evaluation of elliptic curve cryptography and Rivest–Shamir–Adleman cipher suites for high-security and energy-efficient AIoT applications with Fog and Mist computing devices [140]. In [141], Fernández-Caramés et al. developed a Fog Computing-based CPS for the automation of pipe-related tasks. Their system allows pipe tracking and identification, and includes applications based on augmented reality. Salhaoui et al. presented a smart AIoT monitoring and control framework based on UAVs and Fog–Cloud Computing [146]. The framework is validated through a case study to improve product quality and reduce waste in an industrial concrete plant. The proposed architecture implements control strategies and AI-based classification. Jin et al. proposed a collaborative Edge training system for AIoT applications [144]. In order to evaluate and validate the proposed system, they developed two use case examples for smart factory scenarios: a part recognition application and a defective part inspection system. Foukalas et al. developed an AIoT-based Fog Computing application for predictive maintenance in smart factories [143]. For this purpose, AI-based classification and optimization techniques have been implemented. Chu et al. proposed an Edge Computing-based AIoT system for robotic vision guidance in a welding factory [142]. To achieve high-accuracy vision localization, they developed a CNN-based application. These AI techniques are used for production control and optimization tasks. Min et al. presented a machine learning-based digital twin framework for production optimization in the petrochemical industry [139]. They validated the framework through an application to realize intelligent production control based on real-time data. Kuo et al. developed an AIoT-based unmanned vehicle system with a self-learning image recognition algorithm [148].

## 6. Discussion

**Figure 6:**
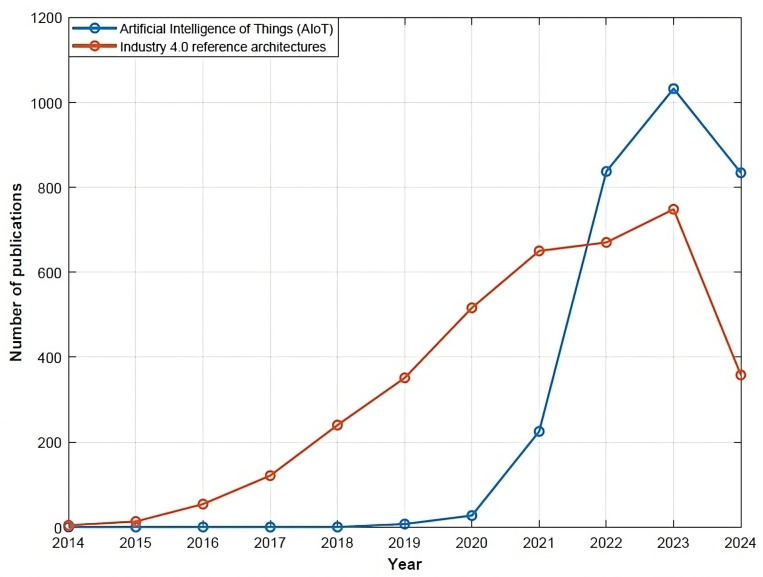
Number of publications in Scopus related to AIoT and Industry 4.0 reference architectures.

The popularity of AIoT has greatly increased in recent years, thanks to the advances in both AI and IoT technologies. Figure 6 shows a graph of AIoT-related publications in the Scopus database in the last 10 years, where a huge increase can be seen since the year 2020. This graph also shows the number of publications in Scopus related to Industry 4.0 reference architectures, which also demonstrates an increasing interest.

**Figure 7:**
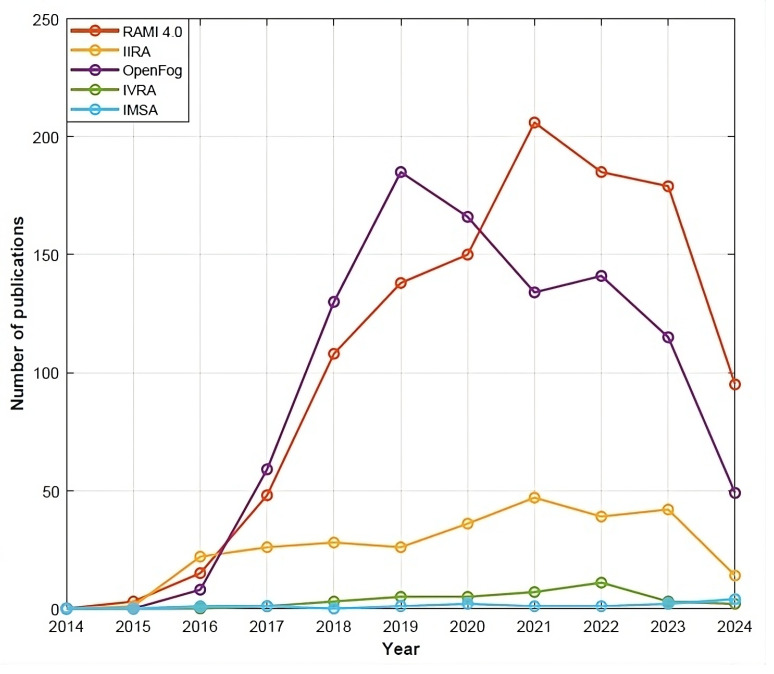
Number of publications in Scopus related to the main reference architectures.

In Section 4, RAMI 4.0, IIRA, OpenFog, IMSA, and IVRA have been identified as the most relevant reference architectures for AIoT applications. Figure 7 shows the number of publications in Scopus related to each one of these reference architectures per year. RAMI 4.0 and IIRA are currently the most mature and popular reference architectures among researchers for industrial applications. OpenFog has also received attention from researchers in recent years. However, although the OpenFog reference architecture can work in Industry 4.0 applications [11], it is a domain-independent architecture that is mainly implemented in other domains such as smart healthcare, smart buildings, and smart cities. IVRA and IMSA are incipient architectures, promoted by the Japanese and Chinese governments, and only a few related studies were found in Scopus. In this research, only English-language works have been analyzed, which might have some impact in the number of studies related to both IVRA and IMSA.

Some reference architectures, like RAMI 4.0, are difficult to understand and require considerable decision making and refinements for implementing them in real industrial applications. The main reason is that they have a high level of abstraction, and sometimes the documentation may be difficult to understand. Thus, these architectures need further development to be implemented in real industrial applications. There is also a clear need for implementation examples that may help designers create new solutions. In this research, several application examples have been analyzed to guide companies choosing the reference architecture that best suits for their application. However, few of these examples provide enough implementation details. For example, Melo et al. provide a deep investigation of the RAMI 4.0 reference model and describe their proposal for the development of an open-source control device for Industry 4.0 applications [71]. However, due to the complexity of RAMI 4.0, this article focuses only on the implementation of a few layers.

Each one of the selected reference architectures has its own challenges and limitations depending on application types and domains. The main distinction between these architectures is that RAMI 4.0, IMSA, and IVRA are domain-specific architectures, which focus on industrial implementations, while IIRA and OpenFog are domain-independent, aiming at different domains, from industry to smart buildings and cities. In order to compare the challenges and guidelines provided by these reference architectures, two features have been selected, digital twin and security. Digital twins have received a lot of attention in recent years, especially in industrial environments. RAMI 4.0, IVRA, and IMSA include some descriptions about their implementation. The IIRA documentation [60] addresses the implementation of digital twins in higher detail, including guidelines and examples to combine digital twins with IIoT. OpenFog is the only architecture that does not include some information about digital twins, because this architecture is more focused on data management and system interoperation than on technological implementation. Regarding security, RAMI 4.0, IMSA, and IVRA mention that security is a crucial part of industrial systems, but they do not provide detailed information about security technologies and implementation guidelines. The IIRA architecture has an additional document that details architectures and best practices to construct trustworthy systems [150]. OpenFog considers security as one of its key pillars and, as such, it identifies the security requirement of the application. However, OpenFog does not clearly describe how to implement security techniques.

Other works propose their own AIoT architectures, which are based neither on reference architectures nor standards. In the following, some representative examples are presented. Liu et al. proposed an AIoT-empowered system architecture dedicated to Edge–Cloud collaborative computing [138]. This article provides an in-detail description of the proposed architecture and investigates enabling technologies for different subsystems of the architecture. The proposed architecture is validated through a real-world CPU/GPU/FPGA-based system implementation and its performance is evaluated. However, this article does not perform research of existing standards and architectures that may suit the proposed application. Neither do authors provide a compatibility analysis with those standards. Ref. [147] presents an industrial augmented reality architecture for the Industry 4.0 shipyard used in Navantia. This work evaluates its performance by means of a real-world use case application. However, the proposed architecture is only briefly detailed, not providing sufficient guidelines or indications about how to implement it in other applications. This article does not mention other existing architectures or standards, especially IoT- and Industry 4.0-related ones, that could be useful for comparing the communication architecture that they propose and explaining the differences with other architectures. Analyzing the rest of the AIoT studies from Section 5, we found that most of them do not implement nor mention the existence of reference architectures that may fit concrete application domains; this was also concluded in [76]. In order to facilitate the implementation of AIoT applications, especially in industry, further research and development of standard reference architectures is needed.

### Challenges and Opportunities

Industry 4.0 introduced new technological advances and structures that can be challenging to implement in most industrial environments. In order to increase the productivity and efficiency of industrial systems, Industry 4.0 systems include a higher number of heterogeneous devices that produce huge amounts of data that need to be analyzed and processed. Industry 4.0 systems are more complex, decentralized, and interconnected. For this reason, Industry 4.0 reference architectures are also broader and cover everything from the business level to the technical issues and product life cycle. These architectures have already made an important contribution, but they still need further development and implementation to facilitate the development of Industry 4.0 systems. For example, there is still no consensus on how companies must deal with legacy industrial systems that need to continue operating. Thus, it requires effort from companies, organizations, and researchers to develop these architectures in order to improve the integration of industrial systems and achieve industry standardization and interconnection.

Reference architectures described in this article are not specifically designed for implementing AI technologies, but their structure offers big opportunities for implementing AI applications. These reference architectures are mainly focused on the integration and interconnection of the subsystems. AI techniques offer great variety and flexibility and can be implemented in different layers of the architecture, depending on the computational capacity. AI technologies may be implemented in a huge amount of application types using simple AI models, for defect detection on products and machines, or more complex models aimed at production scheduling and optimization tasks.

## 7. Conclusions

Implementing Industry 4.0 concepts is still challenging, mainly because of the need to adapt new technologies and paradigms. The introduction of reference architectures may facilitate the development of complex AIoT applications which combine artificial intelligence with IoT technologies. Furthermore, the use of reference architecture standards is essential to achieve better cooperation and compatibility among all application components. However, several reference architectures have been proposed for complex IoT applications. Each of them has some benefits and drawbacks. For example, some of them (RAMI 4.0 and IIRA) are more mature than others (OpenFog, IMSA, and IVRA). In some cases, they address the needs of certain countries (e.g., IMSA and IVRA). Sometimes, e.g., RAMI 4.0, they may use a high level of abstraction that may make their implementation complex in real industrial scenarios. Moreover, unfortunately, only a minority of researchers implement or discuss the compatibility of their proposals with the existing standard reference architectures. This fact limits the long-term validity of their approaches, since they may become particular solutions for specific applications. In this scenario, a review regarding existing standard and non-standard reference architectures for industrial AIoT applications is necessary. In addition, these architectures may become difficult to understand and implement for developers since they introduce several abstract concepts. For this reason, presenting diverse example use cases may help application designers visualize their potential and also offer guidelines and solutions to implement complex Industry 4.0 applications. This article reviews the most common reference architecture standards for developing industrial AIoT systems and surveys application examples, found in the literature, so that developers may choose the alternative that best suits their applications. AIoT application domains are also analyzed in order to identify which are the most popular ones.

Through a systematic review of the scientific literature, five main reference architecture standards have been identified (RQ1), RAMI 4.0, IIRA, OpenFog, IMSA, and IVRA. RAMI 4.0 and IIRA are currently the most relevant reference architecture standards, being promoted by various companies and organizations. Both RAMI 4.0 and IIRA are relatively mature and commonly used for developing new frameworks and solutions. IMSA and IVRA are incipient reference architectures promoted, respectively, by the Chinese and Japanese governments to establish the main concepts and guidelines for the development of their industries. OpenFog is a reference architecture that aims to standardize the implementation of Edge and Fog Computing technologies. It is gaining popularity with the rise of the Fog Computing paradigm in industrial environments. This article also analyzed other, less popular, reference architectures, such as the 5C architecture, LASFA, SITAM, and IoT-RA. Moreover, several vendor-specific reference architectures have been established. One example is the so-called IBM Industry 4.0, which assists in the development of smart manufacturing systems. Standard-based reference architectures play an important role in achieving smart manufacturing standardization. For this reason, architecture alignments and interconnections are being studied by main organizations behind RAMI 4.0, IIRA, and IMSA.

Reference architectures describe abstract concepts that may be difficult to implement. The analysis of diverse application use cases may help engineers create their applications by finding similarities with them. This article presents several application examples of the selected reference architectures (RQ2). A broad number of application examples of RAMI 4.0 and IIRA can be found in the scientific literature, since they are the most mature architectures. Some studies adapt RAMI 4.0 and IIRA architectures for specific application domains. Several OpenFog-based applications can be found in the literature, although most of these applications do not address industrial domains. However, although the adoption of AIoT reference architectures and standards is expected to guide designers in building complex applications, its adoption in the industry is still incipient.

Finally, the article identifies the major application domains where industrial AIoT applications have been deployed (RQ3). Thus, developers may analyze several examples and find out the similarities with their applications.

## Data Availability

Data will be available on request.

## Abbreviations

The following abbreviations are used in this manuscript:

AI	Artificial Intelligence
AIoT	Artificial Intelligence of Things
ANN	Artificial Neural Network
AR	Augmented Reality
CNN	Convolutional Neural Network
CPS	Cyber Physical System
CPPS	Cyber Physical Production System
DL	Deep Learning
IoT	Internet of Things
IIoT	Industrial Internet of Things
ML	Machine Learning
RA	Reference Architecture
RL	Reinforcement Learning
